# Drug induced hepatitis mimicking Wilson’s disease secondary to the use of complex naturopathic regimens: a case report

**DOI:** 10.1186/s12876-019-1122-x

**Published:** 2019-11-27

**Authors:** Tyler Pitre, Jasmine Mah, Jaclyn Vertes, Rosario Rebello, Julie Zhu

**Affiliations:** 1 Michael G. DeGroote School of Medicine (Waterloo Regional Campus), McMaster University, Hamilton, Canada; 2 Faculty of Medicine, Dalhousie University, Halifax, Canada

**Keywords:** Acute liver injury, Naturopathy, Drug-induced liver injury, Rivaroxaban, Wilson’s disease

## Abstract

**Background:**

Drug induced liver injury (DILI) is an important cause of acute liver injury and accounts for approximately 10% of all cases of acute hepatitis. Both prescription and natural health products (NHPs) have been implicated in DILI. There is a dearth of studies on NHPs induced liver injury.

**Case Presentation:**

A previously healthy 37-year-old female presented with subacute hepatitis, in the context of a previous admission to a separate institution, months prior for undiagnosed acute hepatitis. Importantly, she had disclosed taking complex regiments of natural health products (NHPs) for months. Her only other medication was rivaroxaban for her homozygous Factor V Leiden deficiency. She had an extensive work up for causes of acute and unresolving hepatitis. She discontinued several but not all of her NHPs after her initial presentation for acute hepatitis at the first institution and continued taking NHPs until shortly after admission to our institution. The predominant pathological features were that of drug induced liver injury, although an abnormal amount of copper was noted in the core liver biopsies. However, Wilson’s disease was ruled out with normal serum ceruloplasmin and 24-urine copper. After 2 months of stopping all the NHPs, our patient improved significantly since discharge, although there is evidence of fibrosis on ultrasound at last available follow up.

**Conclusion:**

NHPs are a well-established but poorly understood etiology of DILI. The situation is exacerbated by the unregulated and unpredictable nature of many of the potential hepatotoxic effects of these agents, especially in cases of multiple potential toxic agents. This highlights the importance of acquiring a clear history of all medications regardless of prescription status.

## Background

Drug induced liver injury (DILI) is an important cause of acute liver injury, accounting for approximately 10% of all cases of acute hepatitis [1]. DILI has an incidence of 14 to 19 cases per 100,000 persons and it is the most common cause of acute liver injury in the western world, accounting for 50% of cases of acute liver failure [2].

The pathogenesis of DILI is due to a complex interaction of toxic metabolites, immune response and a series of events that starts with intracellular disruption, necrosis, and immunogenic reaction [3]. Injury may be direct hepato-toxicity, through conversion of a xenobiotic substance to an active toxin. Alternatively, the injury may be engendered through immune mechanisms, such as conversion of a cellular protein into an immunogen [3]. Drug reactions may be intrinsic, which means the drug’s effect is predictable and dose dependent such as acetaminophen toxicity. Idiosyncratic reactions are a function of the host’s specific characteristics. For example, certain individuals are more likely to form haptens that act as immunogens and therefore cause an adverse reaction by immune-mediated mechanisms of hypersensitivity [2, 3]. Some drugs can alter P450’s enzymatic activities and heighten the toxicity profile of a co-ingested drug, and these complex interactions can lead to clinically significant DILI. The diagnosis is made typically on a clinical basis, using history and identifying a culpable agent(s). Biopsy in the context of DILI is typically reserved for ruling out competing diagnosis.

Both prescription and natural health products (NHPs) have been found to be associated in DILI [4, 5]. There is a myriad of prescription medications implicated in the development of DILI. For example, of the top ten agents responsible for DILI, nine of them are antimicrobials, with Amoxicillin-Clavulanate topping that list [2].

There are several naturopathic products associated with hepatotoxicity; however, the incidence of those products causing liver injury and the pathogenesis behind them, remains largely unknown [5, 6]. In the United States, the proportion of NHPs causing DILI increased from 7 to 9% in 2004–2007 to 19 to 20% in 2010–2014 [2]. Many of the supplements implicated in DILI are usually combined products instead of individual ones. The most common phenotype for NHPs induced DILI is hepatocellular hepatitis and mixed cholestatic biochemistry, some can lead to fulminant liver failure, requiring liver transplantation [2]. Our case report represents an example of the difficulty of determining the etiology of acute liver injury in the context of multiple NHPs, with uncertain liver toxicity, being taken in conjunction with prescription medications that have known, albeit rare, liver toxicity.

### Case presentation

**Fig. 1:**
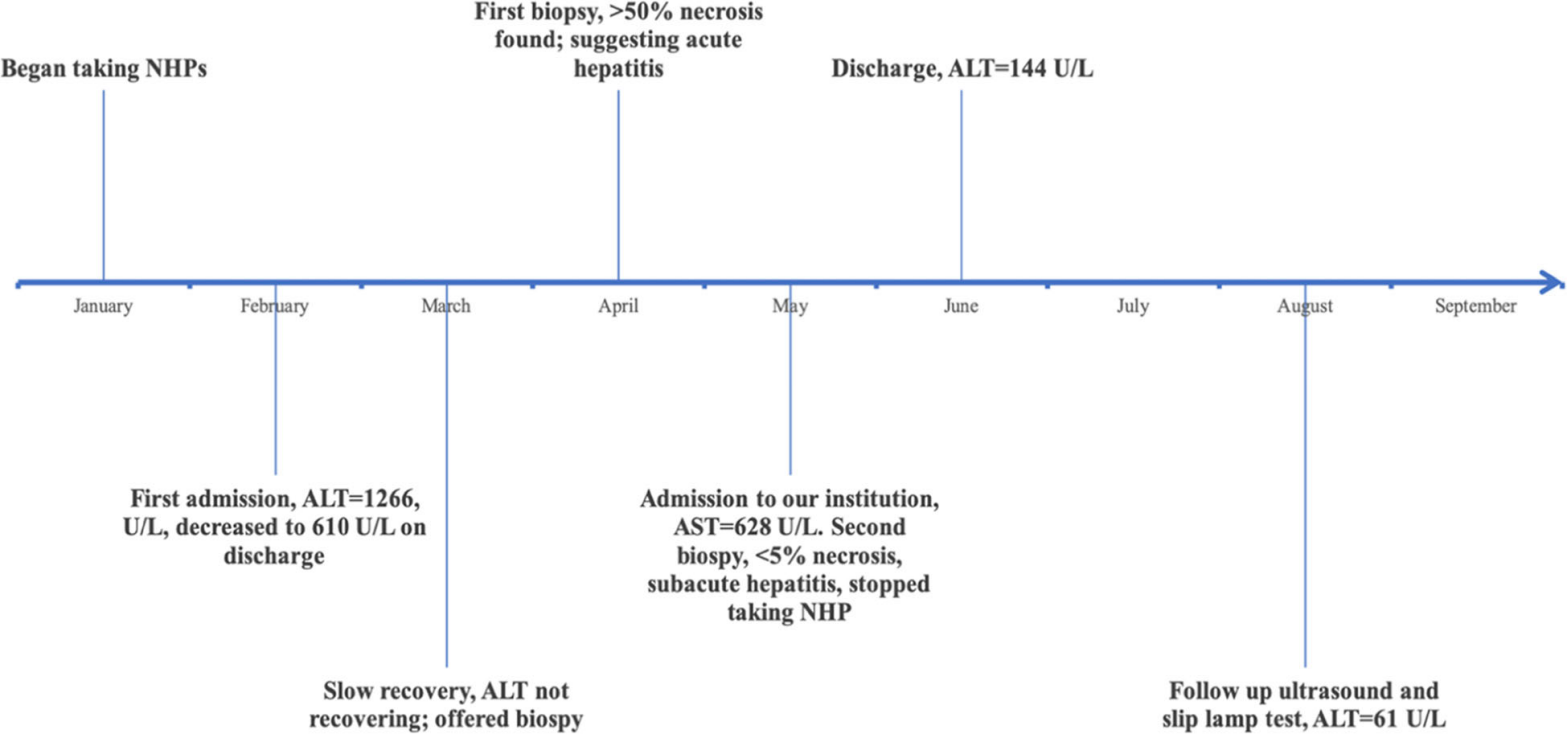
Showing a timeline of our patient’s clinical journey, measured grossly in month intervals. AST is shown as a measure of her slow recovery and final recovery after definitively stopping her NHPs

A previously healthy 37-year-old Caucasian female presented to our institution with a two-month history of jaundice, pruritus and generalized fatigued; having already presented to a different institution for acute hepatitis months prior. Figure 1 highlights a rough timeline of her clinical presentation.

Approximately 1 month prior to her presentation at the first institution, she had treated non-specific abdominal discomfort with various formulations of NHPs through the advice of her naturopathic doctors. Some of her NHPs are summarized in Table 1. She denied any other accompanying symptoms with her prior abdominal discomfort. She noted that 1 day after taking an increasing number of NHPs, she had sudden right upper quadrant pain and dark urine. Notably, this was a different pain than her pre-existing abdominal discomfort. She had a long clinical course at the first institution; she was given a full work up. She was followed in the community on discharge and unfortunately did not have significant improvements in her liver enzymes and clinical status, which led to a first biopsy. She came to us for further management.

**Table 1:** Natural health regimens from January to May 2019

Drug	Dose	Route	Duration	Liver Toxicity Score	Interactions
Ashwagandha	600 mg	PO	5 days	C	None available
Copper	Unknown	PO	1 month	A	No interaction
Ascorbic Acid	3.75–7.25 g	IV	Three treatments	E	No interaction
L-Carnitine	1.25 g–3.75 g	IV	Three treatments	No reports/unknown	No interaction
Acetylcysteine	0.5 g to 2.75 g	IV	Three treatments	E	No interaction
B-Complex 100	0.50 mL to 1.00 mL	IV	Three treatments	No reports/unknown	No interaction
Magnesium Sulfide	0.25 g	IV	Three treatments	No reports/unknown	No interaction
Selenium Chloride	100mcg to 200mcg	PO	BID for 4 months	Cirrhosis in animal studies, no studies in humans	No interaction
Bicarbonate 8.4%	1.25 mL	IV	Three treatments	No reports/unknown	Not available
Rivaroxaban	15 mg	PO	Daily, 2014 to Feb. 2019	B	No interaction
Combined Ingredients of Herbal medications (Herbalife)	N/A	N/A	N/A	A	Not available

Risk defined by Livertox database, which is an evidenced based database from the National Institutes of Health [7]. Drug interactions were tested on all available drugs below using IBM Micromedex drug reference version v2080. A = Well established cause of clinically apparent liver injury. B=Highly likely cause of clinically apparent liver injury. C=Probable cause of clinically apparent liver injury. E = Unlikely cause of clinically apparently liver injury

On presentation to us, the patient was vitally stable and had no evidence of hepatic encephalopathy. She was visibly jaundiced. Abdominal exam was negative for ascites, jugular venous pressure was not elevated but there was mild bilateral pitting pedal edema. There were no stigmata of chronic liver disease. There was no hepatosplenomegaly on exam.

Her past medical history was significant only for homozygous Factor V Leiden deficiency, discovered after an isolated deep vein thrombosis (DVT) diagnosis during pregnancy. For DVT prophylaxis, she was prescribed Rivaroxaban and was taking this medication correctly for years as prescribed. She had no significant travel history, no previous blood transfusion, nor ingestion of undercooked meat to suggest acquisition of hepatitis E. There was no family history of liver disease.

All relevant laboratory findings are summarized in Table 2. Initial laboratory investigations revealed significant transaminitis and decreased synthetic liver function. Her complete blood count was within normal limits. Her viral hepatitis workup was negative; this was inclusive of serology for hepatitis A/B/C/E, EBV and immunoglobulins for CMV infection and HIV antibody/antigen screen was negative. In addition, she had tests for Bartonella, Syphilis, and Malaria smears; ova and parasites tested at the previous institution that were negative. Her metabolic workup was also negative for Hemochromatosis, Wilson’s disease (WD), and alpha-1-antitrypsin deficiency. In addition, she also had a negative antinuclear antibody panel. Other relevant tests included normal an alcohol level and non-detectable acetaminophen level.

**Table 2:** Values of relevant tests performed on admission to our institution and discharge

Test Value	Admission	Discharge	References
AST	967	161	5–45 U/L
ALT	628	144	0–44 U/L
ALP	328	238	38–150 U/L
GGT	182	33	0–49 U/L
Albumin	25	28	35–50 g/L
PTT	48	32	26–38 s
INR	1.6	1.6	0.8–1.2
Total Bilirubin	277.2	357.1	0–20.4 umol/L
Direct Bilirubin	191.9	230.2	0–8.5 umol/L
HIV Ag/Ab screen	Non-reactive		
Variella Zoster IgG	Immune		
Anti-EBNA	Positive*		
CMV IgG	Immune		
HAV IgM	Non-reactive		
HBV			
Hepatitis B e Antibody	Non-reactive		
Hepatitis B e Antigen	Non-reactive		
Surface Ag	Non-reactive		
Core Ab	Non-reactive		
Surface Ab	412.8 mIU/mL (Immune)		
HCV Ab Screen	Non-reactive		
HEPE	Negative serology		
Hemochromatosis			
Cysteine C282Y	Negative		
Histidine H63D	Negative		
Ceruloplasmin	318		200-600 mg/L
Ferritin	221.0		6.5–204 μg/L
Alpha-1-antitrypsin	1.43		1.0–1.9 g/L
Alpha-fetoprotein	7 μg/L		1-8μg/L
Alcohol	Negative		
C-Reactive Protein	2.70 mg/L		
Anti-GBM screen	< 0.2 AI		
Anti-myeloperoxidase	< 0.2 AI		
Anti-proteinase	<0.2 AI		
Anti M-2 Antibodies	Negative		
Anti-SM Antibodies	Negative		
Anti-LKM antibodies	Negative		
ANA panel	* Negative		

*AST* aspartate aminotransferase, *ALT* alanine transaminase, *ALP* alkaline phosphatase, *GGT* gamma-glutamyl transferase, *PTT* partial thromboplastin time, *INR* International normalized ratio, *EBV* epstein-barr virus serology, *CMV IgG* cytomegalovirus Immunoglobulin, *HBV* hepatitis B virus serology, *HEPC* hepatitis C virus serology, *HEPE* hepatitis E virus serology, *ANA* antinuclear antibody, *Anti-LKM* anti-liver-kidney-microsomal antibodies, *Anti-SM antibodies* anti-smooth muscle antibodies, *Anti M-2* anti mitochondrial-2 antibodies. *Anti-GBM* Anti-glomerular basement membrane

*Evidence of past infection

*Including anti-dsDNA, Chromatin, Ribosomal P, SS-A/Ro,SS-B/La, Centromere B, Smooth muscle, Sm/RNP, RNP, Scli-70, Jo-1

**Fig. 2:**
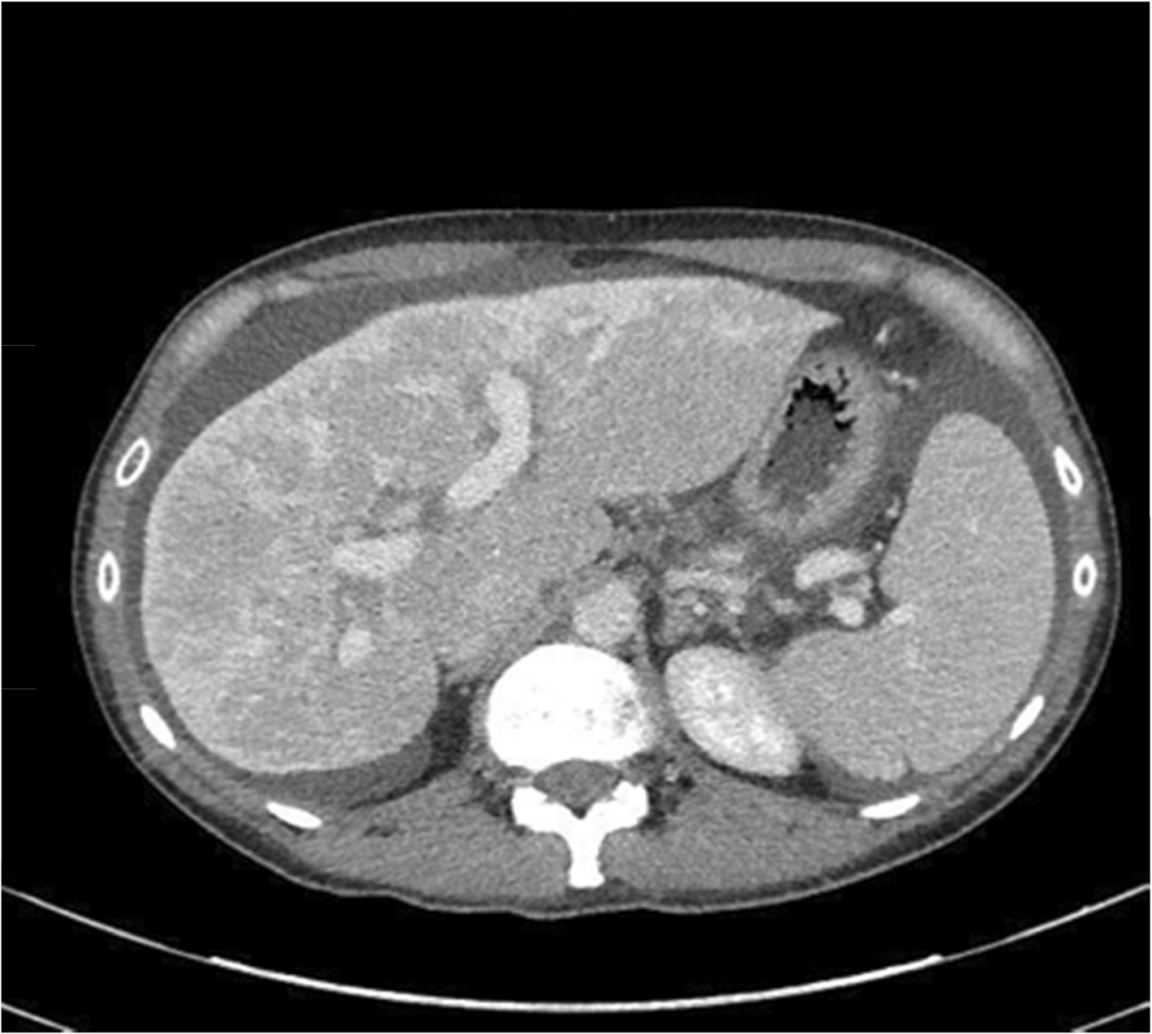
Computed Tomography scan showing unusual parenchymal enhancement with a “flip flop” pattern of reduced enhancement of the peripheral liver in the arterial phase and retention in the portal venous phase

Ultrasound showed a liver with nodularity, with no evidence of hepatic vein or inferior vena cava thrombosis. However, an ultrasound completed months prior in February showed normal hepatic architecture without evidence of cirrhosis. A follow up ultrasound in March revealed a thin rim of fluid around the liver and gallbladder, favoring a reactive cause and interval liver parenchymal edema, consistent with acute hepatitis. She also had a magnetic resonance cholangiography (MRCP) in March, which was also unremarkable. Computed Tomography (CT) studies revealed a lobulated liver contour and lobar redistribution. There was also evidence of portal hypertension with splenomegaly. The liver parenchyma had a nodular morphology, suggestive of regeneration, seen in Fig. 2. The pattern of disease on CT is often seen in Budd Chiari syndrome, but can also be consistent with liver necrosis and regeneration after fulminant hepatitis due to drug toxicity. A biopsy was completed at another institution in April, a month prior to our admission and revealed subacute severe hepatitis with areas of confluent panacinar dropout (about 50% of specimen area affected) with no pathological features of Budd-Chiari. There was also no evidence of fibrosis or cirrhosis from this biopsy. The pathologist’s report suggested drug induced liver injury as a possible diagnosis. Portal hypertension was confirmed by measuring the wedge pressure in the right hepatic vein, showing an elevated hepatic-venous pressure gradient of approximately 14 mmHg (normal is < 5 mmHg).

At our institution, the patient was treated with oral N-acetylcysteine and oral vitamin K 5 mg daily for 3 days and daily ursodiol acids at 13 mg/kg. After 1 week, her synthetic liver function marginally improved but she also developed new clinically relevant ascites. It was revealed that the patient was still taking multiple naturopathic medications including alpha-lipoic acid and magnesium (MgS) and it was recommended that she discontinue these immediately.

During her admission, she was worked up by hepatology for liver transplant candidacy. Fortunately, a repeat trans-jugular core liver biopsy revealed minimal necrosis (less than 5%), with evidence of significant regeneration. Rhodamine staining for copper detected an abnormally high deposition. Again, no evidence of fibrosis or cirrhosis was seen. A diagnosis of drug induced liver injury from NHPs was favored, after she disclosed a significant history of NHPs usage, that had been on-going from her earliest clinical issues until early in her admission to us. She had not disclosed this ongoing usage until the second day of admission to us. She agreed to completely stop taking them.

WD was also considered due the abnormally high level of copper found on the first pathological specimens. However, a 24-h urinary copper and hepatic copper content from her second liver biopsy were subsequently done and were both negative for WD. She also had a negative slip lamp test for Kayser Fleischer rings on last follow up.

She was discharged home with hepatology follow up. In the next 2 months, her liver enzymes and synthetic function progressively normalized and she reports not continuing with any NHPs. The most recent follow up ultrasound reveals a liver with a coarsened echotexture.

### Discussion and conclusion

This case illustrates the difficulty of deriving a precise etiology of acute liver injury in the context of complex, unregulated naturopathic regimens in the background of known liver toxic agents. Direct oral anticoagulants have been known to be hepatotoxic with growing evidence. Ximelagatran, an early factor IIa inhibitor, was withdrawn from the market due to significant hepatotoxicity [7]. Rivaroxaban has been implicated, albeit rarely, in a few case studies and is listed as risk B (highly likely to cause clinically apparent liver injury) by LiverTox [7–9]. However, most of this risk is believed to be during the initial introduction of Rivaroxaban [10]. Meanwhile, our patient had been on Rivaroxaban without adverse effects since 2014, making this unlikely to be the cause of her liver injury.

Acute-on-chronic liver injury was considered, given the evidence of nodular liver on ultrasound, as well as portal hypertension. However, she did not report any signs or symptoms of liver disease until she began taking her NHPs. The previous ultrasounds prior to coming to our institution did not support a diagnosis of underlying cirrhosis. Furthermore, both of her biopsies did not suggest evidence of cirrhosis. Both pathologists separated by time and taken at two different institutions, suggested acute and subacute liver injury as the diagnosis. Portal hypertension occurs typically in the context of cirrhosis but can also happens commonly in acute liver injury [11]. The pathophysiology for portal hypertension in the context of acute liver injury is not well understood but may be due to massive necrosis and reticulin collapse, leading to architectural distortion of hepatic microcirculation [11]. This is consistent with our patient’s biopsy report which revealed massive necrosis and panacinar dropout, which can engender reticulin collapse.

Her ultrasound in June and her follow up ultrasound in August have signs of nodularity and coarse echotexture. Her most available FIB-4 score 2.29, which is not supportive of cirrhosis but does not rule out fibrosis.^1^ Her acute liver injury, due to its prolonged nature, may have caused sufficient architectural distortion to cause fibrosis, which was exacerbated by the patient’s continued taking of her NHPs during her clinical journey, despite advice from her physicians.

A review of the literature was undertaken to explore possible liver outcomes in association with her naturopathic medications. Ashwagandha is a plant root extract, which has a LiverTox profile of C, suggesting a probable cause of apparent liver injury. Vitamin C, even at mega doses, is unlikely to cause clinically significant liver injury. No case reports were found on alpha lipoic acid or probiotics. Our patient was prescribed several regimens of combined intravenous injections which are summarized in Table 1. None of these products have individual high-risk liver toxicity profiles or case reports, but in combination, liver toxicity increases. In addition to these IV treatments, she was prescribed selenium chloride, which has some evidence of causing cirrhosis in animal studies, but no significant liver toxicity has been reported in humans [12]. Finally, there have been multiple case reports on Herbalife, which is a naturopathic medication consisting of a combination of various products [13]. These products have a high LiverTox profile; class A (established cause of clinically apparent liver injury) [9]. Using IBM Micromedex drug reference software, there were no interactions between any of her medications, inclusive of Rivaroxaban and her known NHPs, although some NHPs were not available for comparison (see Table 1).

Complicating the diagnosis was the finding of abnormally high copper levels found on pathology. Upon further questioning, our patient had been ingesting copper in February for approximately 1 month but was unable to recall the dosage. Copper is a well-established cause of acute and chronic liver disease at high doses. Interestingly, our patient’s second biopsy, with the abnormally high copper levels, coincided with a ceruloplasmin level of 318 mg/L and a dry copper weight that was not consistent with WD. As mentioned, our patient had unusual but insufficient copper deposition in her biopsy for a diagnosis of WD.

A case report of a 37-year-old female with acute liver injury in the context of complex natural health products was presented. The association between natural health products and acute liver injury is well established but poorly understood. The combination of various naturopathic medications may lead to more sustained liver injury and hepatotoxicity than ingestion of individual products alone. Our case raises the importance of clinicians inquiring about non-prescription medications in evaluating the potential harmful effects of various naturopathic products.

## Data Availability

The datasets generated and analyzed during the current study are available from the corresponding author on reasonable request.

## Abbreviations

CT: Computed tomography
DILI: Drug induced liver injury
DVT: Deep vein thrombosis
NHPs: Natural Health Products
WD: Wilson’s disease

## Footnotes

1 With AST = 56, ALT = 61, Platelets = 116,000, age = 37.
